# Anti-T-lymphocyte globulin (ATLG) compared to post-transplant cyclophosphamide as GvHD prophylaxis in ALL patients undergoing allogeneic stem cell transplantation

**DOI:** 10.1038/s41409-024-02328-w

**Published:** 2024-06-14

**Authors:** Normann Steiner, Radwan Massoud, Evgeny Klyuchnikov, Nico Gagelmann, Johanna Richter, Christian Niederwieser, Kristin Rathje, Tatjana Urbanowicz, Ameya Kunte, Janik Engelmann, Christina Ihne, Iryna Lastovytska, Cecilia Lindhauer, Franziska Marquard, Mirjam Reichard, Alla Ryzhkova, Rusudan Sabauri, Mathias Schäfersküpper, Niloufar Seyedi, Georgios Kalogeropoulos, Silke Heidenreich, Ina Rudolph, Gaby Zeck, Dietlinde Janson, Christine Wolschke, Francis Ayuk, Nicolaus Kröger

**Affiliations:** 1 Department of Stem Cell Transplantation, University Medical Center Hamburg-Eppendorf, Martinistrasse 52, 20246 Hamburg, Germany; 2 University Hospital of Internal Medicine V (Hematology and Oncology), Medical University of Innsbruck, Anichstraße 35, 6020 Innsbruck, Austria

**Keywords:** Stem-cell research, Leukaemia

## Abstract

We retrospectively analyzed high-risk ALL patients in CR1 receiving total body irradiation based conditioning regimen with ATLG (*n* = 74) or PTCy (*n* = 73) for GVHD prophylaxis. The 3-year OS and LFS were similar in both groups: 65 and 60% in the ATLG group and 64 and 67% in the PTCy group (*p* = 0.9 and 0.5, respectively). CIR and NRM rate at three years was 12 and 21% after PTCy and 19 and 20% after ATLG (*p* = 0.4 and *p* = 0.9, respectively). Acute GvHD grades II-IV and grades III/IV at 100 days was 46 and 19% after PTCy and 33 and 10% after ATLG (*p* = 0.08 and *p* = 0.9, respectively). Chronic GvHD of all grade at two years was higher after PTCy: 55% versus 26% (*p* < 0.001). Based on the propensity score matching (PSM) analysis, aGvHD grades II-IV was trending higher in the PTCy group compared to the ATLG group (*p* = 0.07). In contrast to the PSM analysis, on multivariate analysis the receipt of PTCy compared with ATLG was associated with a reduced CIR (*p* = 0.026). Our retrospective single-center analysis shows a lower incidence of acute and chronic GvHD while displaying similar LFS and OS after ATLG compared to PTCy in TBI based allogeneic stem cell transplantation for high-risk ALL.

## Introduction

Acute lymphoblastic leukemia (ALL) is a hematological malignancy which shows an uncontrolled proliferation of lymphoid progenitor cells and an annual incidence in Europe of about 1.45 per 100,000 individuals [1]. High-risk ALL patients are defined by parameters such as a high white blood cell count, unfavorable cytogenetic abnormalities, older age and late response to induction therapy [2]. Due to improved treatment strategies with implementation of optimized risk stratification and new treatment modalities such as monoclonal antibodies (rituximab), bispecific antibodies (blinatumomab), antibody drug conjugates (inotuzumab-ozogamicin), as well as CART cells, allogeneic stem cell transplantation (alloSCT) still remains the preferred treatment option with a curative intention for high-risk ALL patients in first complete remission [2]. Total Body irradiation (TBI) in combination with cyclophosphamide or etoposide represents a well-established and favored myeloablative conditioning regimen in allogenic transplantation of ALL patients [2–8]. Despite improved conditioning regimens with optimized new immunosuppressive therapies, graft-versus-host disease (GvHD) remains one of the most frequent complication and is associated with a high risk of morbidity and mortality [9]. A preferred GvHD prophylaxis regimen in recent decades in alloSCT patients treated with matched unrelated donor (MUD) is in vivo T cell depletion with anti-T-lymphocyte globulin (ATLG) [10, 11]. In recent years, the GvHD prophylaxis regimen with PTCy has been increasingly used to treat alloSCT patients with haploidentical, MUD or MMUD donors [12–22]. Nevertheless, it remains unclear which T-cell depleting regimen exhibits the highest efficacy. A retrospective study compared PTCy and antithymocyte globulin in AML patients in first complete remission undergoing allogeneic stem cell transplantation from 10/10 HLA-matched donors with similar outcomes [23]. Aydin et al. compared ATG versus PTCy in matched unrelated donors in a non-myeloablative conditioning setting with similar outcomes in both groups [24]. A comparison of PTCy versus ATLG in a considerable ALL cohort in terms of outcome is scarcely to be found in the literature [25, 26]. Therefore, we aimed to compare PTCy and ATLG as GvHD prophylaxis in ALL patients who received a TBI containing conditioning regimen in terms of leukemia-free and overall survival, transplant-related mortality, relapse incidence and incidence of acute and chronic GvHD.

## Patients and methods

### Patients and conditioning strategies

We included 147 adult patients with high-risk ALL in first complete remission (CR1) at the time of conditioning start, independent of MRD status, who received allogeneic SCT from HLA-identical sibling (MRD *n* = 27), matched unrelated donors (MUD *n* = 66), mismatch unrelated donors (MMUD *n* = 41) or haploidentical donors (MMRD *n* = 13) after total body irradiation based conditioning regimen between 1993 and 2023. The database was locked on August 1, 2023. All patients received myeloablative conditioning. A standard conditioning regimen used in our cohort for high-risk ALL patients included TBI 8–12 GY and cyclophosphamide 120 mg/kg actual bodyweight (BW) with or without etoposide, combined with ATLG (Grafalon®, Neovii, Switzerland) in different doses (for MRD 30 mg/kg actual BW, for MUD 60 mg/kg actual BW, and for MMUD 60–90 mg/kg actual BW). We used immunosuppressive agents for GvHD prophylaxis with cyclosporine A (CSA) and mycophenolate mofetil (MMF), or with CyA and methotrexate or with MMF and tacrolimus (details are shown in Table 1). Patients needing reduced immunosuppression [22] were treated with individual approaches with PTCy 50 mg/kg BW on day 3 and day 4 after alloSCT following TBI 12 GY and fludarabine 120 mg/m². Dose reduction using 8 GY TBI was performed in older patients. Our cohort patients transplanted with a MRD received CyA, and patients transplanted with a (M)MUD were given CyA or tacrolimus (mismatch) in combination with mycophenolate mofetil. Moreover, immunosuppressive agents were tapered starting around day 100 until day 120 after alloSCT, depending on clinical presentation and the possible appearance of GvHD. The use of TKIs after alloSCT has not been routinely administered to our patients, but only in cases of molecular relapse. The definition of neutrophil elevation is based on the first 3 consecutive days with a measured absolute neutrophil count ≥0.5 × 10^9^/L. The definition of platelet engraftment includes the first consecutive days with a platelet count ≥20 × 10^9^/L without transfusion support. Acute GvHD was categorized according to the standard criteria [27]. Chronic GvHD was categorized according to the National Institute of Health (NIH) criteria [28]. Patients who developed chronic GVHD before 2005 were reclassified according to the NIH criteria. Disease relapse was defined as a morphological or molecular disease state. On day 100 after alloSCT, the following main outcomes were analyzed: Infections were defined as any positive microbial test that required therapy, CMV and EBV reactivation was defined as the presence of CMV/EBV DNAemia, the diagnosis of hemorrhagic cystitis (HC) was based on the degree of hematuria according to the ECIL guidelines [29], VOD definition was done according to the Baltimore criteria [30], and HSCT-associated thrombotic microangiopathy (TA-TMA) was defined according to the Jodele criteria [31].

**Table 1:** Patient, donor and transplant characteristics.

	ATLG	PTCY	*p*
	N (%)	N (%)	
Patient age median (range)	29 (18–61)	42 (18–79)	**<0.001**
Age ≤36	49 (66)	24 (33)	**<0.001**
Age >36	25 (34)	49 (67)	
ALL			**0.02**
B	49 (66)	64 (88)	
T	25 (34)	9 (12)	
BCR-ABL			**0.006**
–	14 (935)	40 (63)	
+	26 (65)	24 (38)	
MRD at transplant			0.38
–	10 (48)	38 (59)	
+	11 (52)	27 (42)	
SCT year median (range)	2003 (1993–2015)	2020 (2014–2023)	**<0.001**
SCT ≤ 2014	72 (37)	2 (3)	**<0.001**
SCT > 2014	2 (3)	71 (97)	
Type of donor			**0.006**
MRD	9 (12)	18 (25)	
MMRD	3 (4)	10 (14)	
MUD	34 (46)	32 (44)	
MMUD	28 (38)	13 (18)	
Patient / Donor Sex			0.06
M/M	32 (43)	28 (38)	
M/F	13 (18)	7 (10)	
F/F	18 (24)	14 (19)	
F/M	11 (15)	24 (33)	
Patient / Donor CMV			0.5
–/–	22 (30)	19 (26)	
–/+	12 (16)	7 (10)	
+/+	27 (37)	34 (47)	
+/–	13 (18)	13 (918)	
ABO incompatibility			0.073
Compatible	29 (39)	44 (60)	
Minor incompatibility	19 (26)	11 (15)	
Major incompatibility	18 (24)	11 (15)	
Bidirectional incompatibility	8 (11)	7 (10)	
Donor Age median (range)	40 (15–65)	30 (18–72)	**<0.001**
Age ≤37	34 (46)	44 (60)	0.082
Age >37	40 (54)	29 (40)	
Stemcell source			**<0.001**
BM	28 (38)	8 (11)	
PBSC	46 (62)	65 (89)	
CD34 infused cells median (range)	6.6 (0.6–31)	6.8 (1.5–11.4)	0.8
≤6.7 × 10^6^/kg BW	38 (51)	36 (49)	
>6.7 × 10^6^/kg BW	36 (49)	37 (51)	
Conditioning			**<0.001**
TBI+Cy+Vp16	52 (70)	0 (0)	
TBI+Cy	22 (30)	0 (0)	
TBI+Flu	0 (0)	73 (100)	
TBI dose			**<0.001**
8.0	0 (0)	22 (30)	
10.0	1 (1)	1 (1)	
12.0	73 (99)	50 (69)	
ATLG dose			
30.0	11 (15)		
40.0	1 (1)		
60.0	29 (39)		
90.0	26 (35)		
other	7 (10)		
Immune suppression			**<0.001**
MTX + CSA	50 (67)	0 (0)	
MMF + CSA	22 (30)	35 (48)	
MMF + TAC	0 (0)	24 (33)	
Unknown	2 (3)	14 (19)	

Bold values indicate statistical significance *p*  <  0.05.

*ALL* acute lymphoblastic leukemia, *ATLG* anti-t-lymphocyte globulin, *PTCy* post- transplant cyclophosphamide, *SCT* stem cell transplantation, *MRD* minimal residual disease, *MRD* matched related donor, *MMRD* mismatch related donor, *MUD* matched unrelated donor, *MMUD* mismatched unrelated donor, *CMV* cytomegalo virus, *BM* bone marrow, *PBSC* peripheral blood stem cell, *Cy* cyclophosphamide, *Vp16* etoposide, *Flu* fludarabine, *TBI* total body irradiation, *CSA* cyclosporine A, *MMF* mycophenolate mofetil, *MTX* methotrexate, *TAC* tacrolimus.

### Ethics approval and consent to participate

The study was approved by the institutional review board and conducted in accordance with the Declaration of Helsinki and the guidelines for good clinical practice. All patients gave written informed consent.

### Study endpoints

The primary endpoints of this retrospective single-center analysis were overall survival (OS), leukemia-free survival (LFS), and GvHD relapse-free survival (GRFS). Secondary endpoints were non-relapse mortality (NRM), relapse incidence (RI), and incidence of acute and chronic GvHD.

### Statistical methods

Data were retrospectively reviewed and analyzed as of August 2023. Calculations were performed with the Statistical Program for the Social Sciences (SPSS), version 22 (IBM, Armonk, NY, USA), and for competing risk analyses the cmprsk package in R version 4.2.2 [32]. Continuous variables were compared with the Mann-Whitney U test and categorical variables with the Chi² test and for small patient numbers Fisher’s Exact test was used. Overall survival (OS) and leukemia-free survival (LFS) were calculated using the Kaplan-Meier method. For calculation of OS, death from any cause was considered an event and surviving patients were censored at last follow-up. For calculation of LFS, relapse or death from any cause was considered an event and surviving patients were censored at last follow-up. GRFS events were defined as grade 3 or 4 acute GvHD, chronic GvHD (moderate or severe) requiring systemic immunosuppressive substances, disease relapse, or death from any cause after alloSCT. The log-rank test was performed to evaluate differences between individual curves. A cumulative incidence function was performed to calculate the incidence of relapse and of non-relapse mortality, viewing the two as competing risks [33]. The cumulative incidence of infections, CMV reactivation, HC, VOD, and TMA were calculated in a competing risk setting with death without occurrence of the event as a competing event. The cumulative incidence of aGvHD and cGvHD were calculated in a competing risk setting with death, graft failure (GF) as competing events for both and additionally cGvHD as a competing event for aGvHD. Multivariate analysis was calculated using a Cox proportional-hazards model and different factors were included in the Cox model. All tests were two-sided, statistical significance was defined as *p* < 0.05 [34]. In this study, we implemented propensity score matching to balance covariates between the ATLG and PTCY groups [35]. Propensity scores were estimated using Firth’s penalized logistic regression to address perfect separation in the female-into-male allografting versus other variable, which is a significant predictor of acute and chronic GvHD. The logistic regression model included the following covariates: patient age, disease classification (B-ALL vs. T-ALL), donor type (related versus unrelated), patient sex, donor age, stem cell source, and female-into-male allografting (female into male versus other). Nearest neighbor matching was performed with a caliper set to 0.1 times the standard deviation of the logit-transformed propensity scores to ensure an adequate balance between the treatment groups. The effectiveness of the matching process was evaluated by calculating absolute standardized mean differences (SMDs) and variance ratios for each covariate before and after matching. Balance improvement was visually confirmed through density plots of the propensity score distributions and SMD plots.

The correlation among key variables was assessed to detect multicollinearity. The variables of interest, including ATLG versus PTCy, conditioning regimen and year of transplant were selected from the dataset. A correlation matrix was then computed using the cor() function in R. This matrix provided insight into the relationships between variables. Additionally, a visually correlation matrix was generated using the corrplot package for better interpretation of the results.

## Results

### Patient, donor and transplant characteristics

A total of 147 patients in CR1 were included in our retrospective analysis covering the period from 1993 to 2023, of whom 74 patients were treated with ATLG and 73 patients with PTCy as part of immunosuppressive therapy. Patients in the PTCy group were older (median age 42 vs 29 years in the ATLG group; *p* < 0.001). In the PTCy group, 22 patients were conditioned using 8 Gy TBI, one patient with 10 GY TBI and 50 patients using 12 GY TBI. In the ATLG group one patient received 10 GY TBI and 73 patients received 12 GY TBI. Cell count of CD34 x10^6^ /kg BW did not differ between PTCy and ATLG. Two patients in the ATLG group died before engraftment. All other patients in both groups showed leukocyte engraftment. The platelet engraftment rate was 90 and 92% in the ATLG and PTCY group respectively (*p* = 0.7). A detailed list of patients and donor characteristics is given in Table 1.

### Toxicity

At day 100, the incidence of cystitis was greater in the PTCy group with 29% of patients versus 16% in the ATLG group (*p* = 0.24). A significantly higher rate of veno-occlusive disease (VOD) at day 100 was observed in the PTCy group, namely 10% of patients versus 3% of patients in the ATLG group (*p* = 0.04). Transplant associated thrombotic microangiopathy (TMA) occurred at a significantly higher rate at day 100 in the PTCy group, namely 5% of patients versus 2% of patients in the ATLG group (*p* = 0.03). Infections at day 100 were observed in 68% of patients in the PTCy group versus 56% in the ATLG group (*p* = 0.0012). CMV reactivations at day 100 were observed in higher numbers in the PTCy group with 53% of patients versus 31% of patients in the ATLG group (p = 0.0002). This difference was also evident in the subgroup analysis of patients with positive CMV serology at transplant, where the CMV reactivation incidence was 47% in the ATLG group and 69% in the PTCy group (*p* = 0.008). EBV reactivation at day 100 was lower in the PTCy group, namely 10% versus 21% in the ATLG group (*p* = 0.1).

### Graft-versus-host disease

**Fig. 1:**
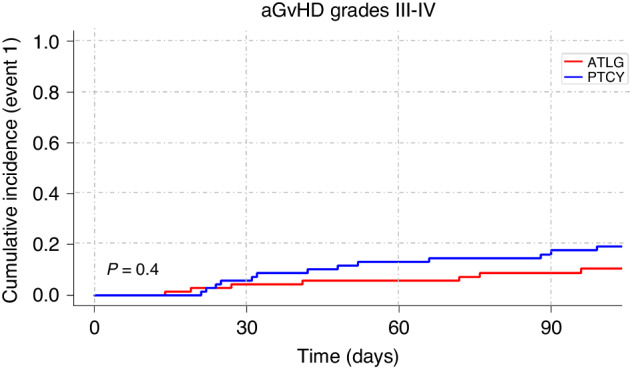
Cumulative incidence of acute GvHD grades III–IV after alloSCT for ALL patients treated with ATLG or PTCy. Comparison of the cumulative incidence of grades III–IV aGvHD over a 100-day period in ALL patients treated with ATLG or PTCy.

**Fig. 2:**
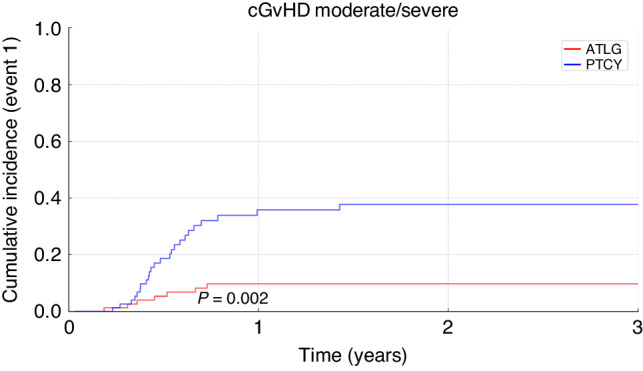
Cumulative incidence of chronic GvHD moderate/severe after alloSCT for ALL patients treated with ATLG or PTCy. Comparison of cumulative incidence of moderate and severe cGvHD over a 3-year period in ALL patients treated with ATLG or PTCy.

Cumulative incidence of acute GvHD grades II-IV at 100 days was 33% (95%CI 22%–44%) in the ATLG group versus 46% (95%CI 34%–58%) in the PTCy group (*p* = 0.08). Cumulative incidence of acute GvHD grades III/IV at 100 days was 19% (95%CI 11%–29%) after PTCy and 10% (95%CI 4%–18%) after ATLG (*p* = 0.4; Fig. 1). A significantly higher rate of chronic GvHD was seen in the PTCy group than in the ATLG group (*p* < 0.001) with a corresponding two-year cumulative incidence of chronic GvHD of all grades of 26% (95%CI 17%–37%) in the ATLG group versus 55% (95%CI 42%–67%) in the PTCy group (*p* < 0.001). Cumulative incidence of chronic GvHD (moderate and severe) at two years was 9% (95%CI 4%–17%) in the ATLG group versus 36% (95%CI 25%–48%) in the PTCy group (*p* = 0.002; Fig. 2).

### Non-relapse mortality

Non-relapse mortality (NRM) at three years was 21% (95%CI 12%-31%) in the PTCy group compared to 20% (95%CI 12%-30%) in the ATLG group (*p* = 0.9). In the univariate analysis, the cumulative incidence of NRM was higher for patients > 36 years compared to ≤ 36 years (at 3 years 28% versus 13%; *p* = 0.03; Table 2). On multivariate analysis, patient age (HR 1.021 [95%CI 1–1.04] *p* = 0.046) was associated with significantly higher NRM rate (Table 3).

**Table 2:** Univariate analysis.

Univariate analysis	3 y GRFS	3 y NRM	3 y REL	3 y OS	3 y LFS
	*p*	*p*	*p*	*p*	*p*
SCT year	0.4	0.85	0.15	0.6	0.3
SCTyear ≤2014	42%	20%	16%	64%	58%
SCTyear > 2014	36%	21%	10%	65%	69%
ATLG vs PTCY	0.22	0.86	0.4	0.9	0.54
ATLG	43%	20%	19%	65%	60%
PTCY	34%	21%	12%	64%	67%
Match vs Mismatch	0.9	0.55	0.64	0.39	0.4
Match	38%	19%	13%	68%	65%
Mismatch	39%	22%	14%	60%	59%
Patient/Donor SEX	0.9	0.31	0.46	0.38	0.36
M/M	39%	19%	19%	63%	60%
M/F	35%	31%	5%	64%	58%
F/F	35%	25%	17%	58%	58%
F/M	43%	12%	6%	81%	74%
Patient/Donor CMV	0.3	0.9	0.05	**0.021**	0.08
neg/neg	44%	20%	5%	77%	71%
neg/pos	49%	21%	0%	79%	72%
pos/pos	36%	18%	17%	64%	63%
pos/neg	25%	24%	30%	36%	36%
ABO incompatibility	0.47	0.47	0.18	0.69	0.67
Compatible	39%	24%	9%	67%	65%
Minor incomp.	41%	20%	20%	62%	60%
Major incomp.	46%	14%	11%	70%	67%
bidirectional incomp.	20%	13%	27%	53%	53%
Patient age	**0.032**	**0.03**	0.99	**0.021**	**0.041**
≤36	49%	13%	13%	74%	72%
>36	28%	28%	15%	54%	53%
Donor age	0.33	0.87	0.09	0.28	0.29
≤37	37%	19%	19%	64%	61%
>37	40%	22%	8%	66%	65%
Stemcell source	0.9	0.5	**0.03**	**0.03**	**0.018**
BM	40%	25%	25%	50%	47%
PBSC	38%	19%	10%	70%	68%
CD34 infused cells	0.88	0.14	0.45	0.05	**0.049**
≤6.7	38%	26%	15%	56%	53%
>6.7	39%	14%	12%	75%	72%
B-ALL vs T-ALL	0.97	0.59	0.69	0.6	0.81
B-ALL	38%	21%	13%	63%	62%
T-ALL	41%	17%	15%	70%	65%
BCR-ABL	0.54	0.89	0.49	0.89	0.7
Neg	31%	24%	12%	60%	59%
Pos	37%	22%	13%	64%	60%
MRD	0.8	0.33	0.77	0.37	0.4
Neg	39%	20%	9%	69%	69%
Pos	36%	14%	11%	74%	70%
Conditioning	0.43	0.65	0.17	0.5	0.24
TBICyVP16	44%	23%	17%	62%	54%
TBICy	41%	14%	9%	73%	73%
TBIFlu	34%	21%	12%	64%	60%
TBI dose	0.32	0.61	**0.02**	0.22	0.23
<12 Gy	28%	18%	31%	50%	50%
12 Gy	40%	21%	10%	68%	65%

Bold values indicate statistical significance *p*  <  0.05.

*3* *y* 3 years, *OS* overall survival, *LFS* leukemia-free survival, *GRFS* GvHD and relapse-free survival, *CIR* cumulative incidence of relapse, *NRM* non-relapse mortality, *ALL* acute lymphoblastic leukemia, *ATLG* anti-t-lymphocyte globulin, *PTCy* post- transplant cyclophosphamide, *SCT* stem cell transplantation, *MRD* minimal residual disease, *CMV* cytomegalo virus, *BM* bone marrow, *PBSC* peripheral blood stem cell, *Cy* cyclophosphamide, *VP16* etoposide, *Flu* fludarabine, *TBI* total body irradiation.

**Table 3:** Multivariate analysis.

Variable	OS	LFS	GRFS
	HR [95% CI]	HR [95% CI]	HR [95% CI]
	*p*	*p*	*p*
Patient age (1year increase)	**1.02 [1.01–1.04] 0.007**	**1.03 [1.01–1.04] 0.003**	1.01 [0.99–1.02] 0.5
PTCY vs. ATLG	0.69 [0.35–1.36] 0.3	0.62 [0.32–1.19] 0.2	1.21 [0.66–2.23] 0.5
HLA Mismatch vs Match	1.17 [0.65–2.09] 0.6	1.17 [0.67–2.06] 0.6	0.95 [0.53–1.70] 0.9
Patient CMV serology pos vs neg	1.53 [0.81–2.89] 0.2	1.28 [0.71–2.33] 0.4	1.38 [0.78–2.44] 0.3
PBSC vs. BM	**0.5 [0.27–0.91] 0.023**	0.48 [0.27–0.87] 0.015	1.02 [0.40–2.58] 0.9

	CIR
	HR [95% CI]
	*p*
Donor age (1 year increase)	0.991 [0.9596–1.023] 0.580
PTCY vs ATLG	**0.268 [0.0841–0.851] 0.026**
Source [PBSC vs BM]	**0.413 [0.1733–0.986] 0.046**
TBI [12 Gy vs <12 Gy]	**0.121 [0.0362**–**0.406] 0.00063**
	**NRM**
	**HR [95% CI]**
	***p***
Patient age (1 yr increase)	**1.021 [1.000–1.040] 0.046**
PTCY vs ATLG	0.809 [0.321–2.040] 0.650
Source [PBSC vs BM]	0.685 [0.299–1.570] 0.370

Bold values indicate statistical significance *p*  <  0.05.

*OS* overall survival, *LFS* leukemia-free survival, *GRFS* GvHD and relapse-free survival, *CIR* cumulative incidence of relapse, *NRM* non-relapse mortality, *ATLG* anti-t-lymphocyte globulin, *PTCy* post- transplant cyclophosphamide, *P/D* patient/donor, *CMV* cytomegalo virus, *BM* bone marrow, *PBSC* peripheral blood stem cell, *TBI* total body irradiation.

### Cumulative incidence of relapse

Cumulative incidence of relapse (CIR) at three years was 12% (95%CI 5%-21%) in the PTCy group versus 19% (95%CI 11%-29%) in the ATLG group (*p* = 0.4).

Only stem cell source (3 years CIR BM 25% versus 10% PBSC, *p* = 0.03), and TBI dose (3 years CIR < 12 Gy 31% versus 10% 12 Gy, *p* = 0.02) affected CIR in univariate analysis (Table 2). On multivariate analysis, PTCy versus ATLG (HR 0.268 [95%CI 0.0841–0.851] *p* = 0.026), PBSC versus BM (HR 0.413 [95%CI 0.1733–0.986] *p* = 0.046) and the TBI dose of 12 Gy versus <12 Gy (HR 0.121 [95%CI 0.0362–0.406] *p* = 0.00063) were associated with decreased CIR (Table 3).

### Leukemia-free survival

**Fig. 3:**
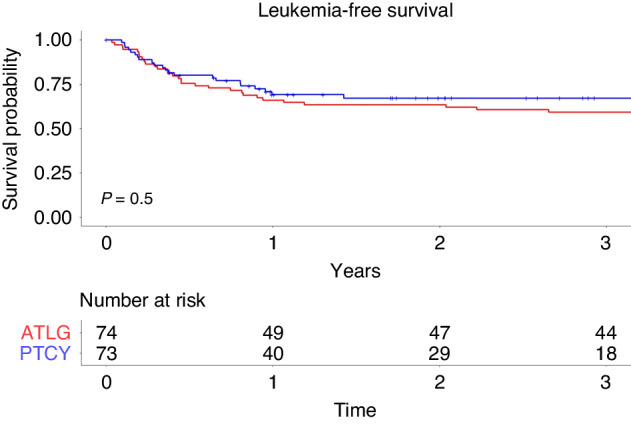
Leukemia-free survival after alloSCT for ALL patients treated with ATLG or PTCy. Comparison of leukemia-free survival in ALL patients treated with ATLG or PTCy over a 3-year period.

Regarding leukemia-free survival (LFS), no significant differences were seen in the two groups. LFS at three years in the PTCy group was 67% (95%CI 57%–80%) versus 60% (95%CI 49%–72%) in the ATLG group (*p* = 0.5; Fig. 3). We observed improved LFS for younger patients (at 3 years ≤ 36 years 72% versus 53% > 36 years; *p* = 0.041), for patients receiving PBSC compared to bone marrow (68% versus 47%; *p* = 0.018) and for patients who received >6.7 × 10^6^ CD34 infused cells/ kg body weight compared to ≤6.7 × 10^6^ cell/kg body weight (72% versus 53%; *p* = 0.049; Table 2). On multivariate analysis, only patient age (HR 1.03 [95%CI 1.01–1.04]; *p* = 0.003) was associated with significant effect on leukemia free survival (Table 3).

### Overall survival

**Fig. 4:**
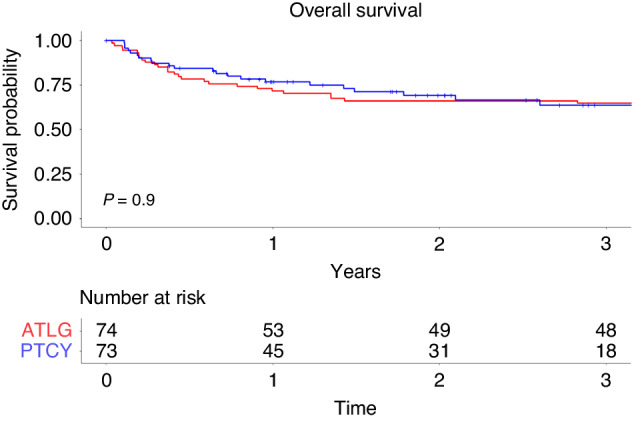
Overall survival after alloSCT for ALL patients treated with ATLG or PTCy. Comparison of overall survival in ALL patients treated with ATLG or PTCy over a 3-year period.

Between the PTCy and the ATLG cohorts no significant difference was seen regarding overall survival (OS). OS at three years was 64% in the PTCy group [95% CI 52%–78%] and 65% in the ATLG group [95% CI 54%–76%]; *p* = 0.9 (Fig. 4). In the univariate analysis, younger patients (p = 0.021), CMV serology (*p* = 0.021), and stem cell source (*p* = 0.03) significantly affected overall survival (Table 2). On multivariate analysis, only patient age (HR1.02 [95%CI 1.01–1.04]; *p* = 0.007) and stem cell source PBSC versus BM (HR 0.5 [95%CI 0.27–0.91]; *p* = 0.023) significantly affected overall survival (Table 3).

### GvHD relapse-free survival

**Fig. 5:**
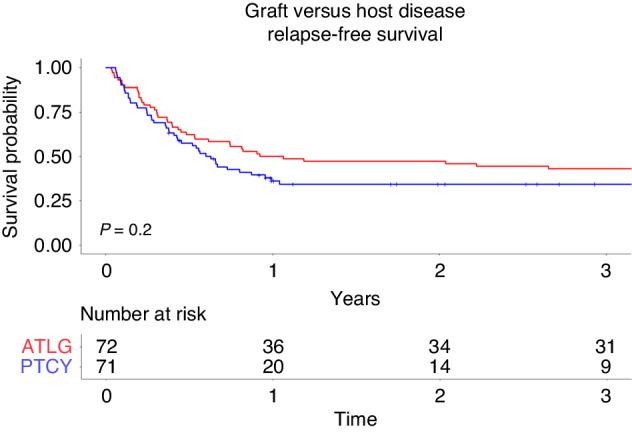
GvHD relapse-free survival after alloSCT for ALL patients treated with ATLG or PTCy. Comparison of relapse-free survival in ALL patients treated with ATLG or PTCy over a 3-year period.

GvHD relapse-free survival at three years in the PTCy group was 34% [95%CI 25%-48%] versus 43% [95%CI 33%-56%] in the ATLG group (*p* = 0.2; Fig. 5). In the univariate analysis patients with ≤36 years had a significantly higher GvHD relapse-free survival compared to patients >36 years (49% versus 28%, *p* = 0.032; Table 2). None of the factors significantly affected GRFS on multivariate analysis (Table 3).

### Propensity score matching and colinear

Due to the heterogeneous groups we conducted beside multivariate analysis also a propensity score analysis. The implementation of propensity score matching significantly improved the balance between the ATLG and PTCy groups across multiple covariates. Before matching, substantial imbalances were observed in variables such as propensity score (SMD = 1.5328) and patient age (SMD = −0.9731). After matching, the standardized mean differences for these covariates were notably reduced, with the propensity score SMD dropping to 0.0434 and patient age SMD to −0.2462. All other covariates exhibited SMDs close to zero post-matching, indicating effective balance. The variance ratios for continuous variables like donor age also approached 1, further validating the balance improvement. The propensity score distribution plots confirmed that the matching process effectively aligned the distributions of the treated and control groups (Supplementary Fig. 1). Additionally, the SMD plot illustrated a substantial reduction in imbalances for all covariates, demonstrating the robustness of the matching process in achieving comparable treatment groups (Supplementary Fig. 2). The matching process resulted in 31 treated patients being matched with 31 control patients.

Based on the propensity score matching analysis, we observed a trend for higher incidence of aGvHD grades II-IV (HR 1.99 [95%CI 0.95–4.16], *p* = 0.07) in the PTCy group compared to the ATLG group, with no significant differences observed in other transplant outcomes (Supplementary Fig. 3).

A correlation analysis was conducted, revealing high correlations between the variables ATLG versus PTCy, type of conditioning and year of transplant, female transplanted into male.

### Sub-analysis of transplant outcomes of MRD positive versus MRD negative patients

Sub-analysis of 11 MRD positive/10 MRD negative patients in the ATLG group and of 27 MRD positive/38 MRD negative patients in the PTCy group. Regarding overall survival, leukemia-free survival, GvHD relapse-free survival, relapse incidence, and NRM no significant differences were seen between the two groups (Table 2).

## Discussion

This retrospective single-center analysis of high-risk ALL patients showed that TBI-based alloSCT with PTCy as GvHD prophylaxis leads to similar LFS and OS in comparison to ATLG but is associated with higher incidence of acute and chronic GvHD and infectious complications.

Giebel et al. reported on behalf of EBMT 2-year OS rates for ALL patients treated with alloSCT in CR1 between 60% (46–55-year-old patients transplanted from MSD/MUD) and 76% (18–25-year-old patients transplanted from MSD) with a relapse incidence of up to 26% and a non-relapse mortality of 29% at two years after transplantation [3].

Allogeneic SCT plays an important role and is probably the only curative treatment strategy to date, even though it involves the risk of GvHD. Despite improved conditioning regimen and optimized GvHD prophylaxis, patients still die from complications of severe GvHD. Therefore, evaluation of different GvHD prophylaxis is urgently needed.

In Europe, the prevailing therapeutic approach to prevent GvHD includes standard prophylaxis comprising CNIs, MTX, or mycophenolate mofetil in combination with one of the available rabbit ATGs for unrelated donor transplantation, and in recent years for siblings as well [36]. There are currently two types of rabbit ATGs, which consist of polyclonal IgG obtained from the hyperimmune sera of rabbits. These IgG antibodies are immunized either with human thymocytes in the case of ATG (anti-thymocyte globulin, Thymoglobulin), or with human Jurkat leukemia T-cell lines in the case of ATLG (anti-T-lymphocyte globulin, Grafalon) [37].

In a meta-analysis from 2017, we described that ATLG is most likely the better option for preventing chronic and acute GVHD in the context of allogeneic stem cell transplantation compared to thymoglobulin. We also reported that ATLG and Thymoglobulin have similar efficacy in terms of TRM [38].

In recent years, in addition to a well-established GvHD prophylaxis regimen with ATG/ATLG, which is widely used in Europe, PTCy-based strategies have become part of conditioning regimens [19–22, 39–50]. Bailen et al. observed no significant differences in OS, EFS, CIR, NRM and GRFS with PTCy or ATG based GvHD prophylaxis in a retrospective study with 132 heterogenous patients (AML, MDS, ALL, NHL, CLL and others) undergoing a matched or 9/10 mismatched unrelated donor SCT. There was a lower incidence of grades II-IV and III-IV aGvHD at 100 days in the PTCy group compared to the ATG group (*p* = 0.008 and *p* = 0.003 respectively). No significant differences in the two-year cumulative incidence of moderate-severe chronic GvHD were observed between the two groups [41]. Van Gorkom et al. analyzed CLL patients receiving a haploidentical SCT with PTCy as GvHD prophylaxis and showed, despite the use of PTCy, a relapse incidence not higher than after HLA matched SCT [42]. Santoro et al. described in 208 ALL patients receiving an unmanipulated haploidentical allo-SCT with PTCy or ATG based GvHD prophylaxis, for patients in CR1 a OS, LFS, and GRFS at 3 years with 52%, 47%, and 40% respectively. Cumulative incidence of grades II-IV aGvHD was 31%, grades III-IV 11% and chronic GvHD 29% with a NRM rate of 32% and a CIR of 37% [43]. In a retrospective study by Ruggeri et al. of 308 AML patients receiving PTCy as GvHD prophylaxis in a haploidentical setting, better LFS and GRFS and lower GvHD and NRM rates were observed compared to patients receiving ATG [44]. A report by the EBMT pictured for AML and ALL patients receiving a T-replete haploidenical SCT with PTCy based regimen for GvHD prophylaxis in 25% in the RIC and in 32% in the MAC group this regimen, in particular associated with PTCy, as a valid option in first line therapy of high risk AML or ALL patients [45]. Jimenez Jimenez revealed a superior GRFS and OS in 128 patients with acute leukemia, MDS, and NHL receiving PTCy based GvHD prophylaxis compared to ATG after HLA-mismatched unrelated donor transplant. Moreover, allograft recipients of ATG-based prophylaxis had a higher NRM rate compared to patients receiving PTCy [46].

A further EBMT work of Battipaglia et al. results in AML patients with HLA-mismatched unrelated donor transplantation (9/10 MMUD) receiving PTCy as GvHD prophylaxis in a lower incidence of severe aGvHD and a better survival compared with ATG patients [47].

Nykolyszyn et al. analyzed lymphoid and myeloid patients receiving PTCy or ATG as GvHD prophylaxis for mismatched unrelated SCT. The authors demonstrated a significantly lower rate of grades II to IV aGvHD in the PTCy group compared to the ATG group (*p* = 0.002). No differences were observed in the cumulative incidence of cGvHD. The NRM rate and CIR were significantly lower in the PTCy group than in the ATG group (*p* = 0.021 and *p* = 0.07 respectively), PFS, OS and GRFS were significantly better in the PTCy group than in the ATG group (*p* = 0.006, p = 0.026 and *p* = 0.011 respectively) [48]. A retrospective work by Mehta et al. dealt with 113 patients with high risk hematological malignancies receiving PTCy or conventional based GvHD prophylaxis with ATG in mismatched unrelated SCT. The incidence of grades II-IV and grades III-IV aGvHD was similar at day 100 between the two groups. Chronic GvHD, two-year NRM, CIR, PFS or OS were also similar between the two groups [49]. Moiseev et al. showed a significantly lower cumulative incidence of grades II to IV acute GvHD (*p* = 0.0003), grades III to IV acute GvHD (*p* < 0.0001) and chronic GvHD (p < 0.0001) in the PTCy group compared to the ATG group in AML and ALL patients receiving PTCy- or ATG-based GvHD prophylaxis in the setting of unrelated peripheral blood stem cell transplantation. PTCy-based GvHD prophylaxis was associated with reduced NRM (*p* = 0.005) and improved OS (*p* = 0.0007), EFS (*p* = 0.0006) and GRFS (*p* < 0.0001). Patients receiving PTCy-based prophylaxis experienced less VOD, CMV reactivation, invasive mycosis and lower grades of mucositis compared to patients receiving ATG [50]. Reasons for all these varying results could be the inclusion of different hematologic diseases or heterogeneous composition within the groups. Brissot et al. observed similar outcomes in AML patients in first complete remission undergoing allogeneic stem cell transplantation from 10/10 HLA-matched donors with two different conditioning regimens and no significant differences between PTCy and ATG for the incidence of grades II-IV acute GVHD or for chronic GVHD or extensive chronic GVHD [23]. Aydin et al. demonstrated a significantly lower rate of acute GvHD grades II-IV for the PTCy-based regimen in their analysis in matched unrelated donors in a non-myeloablative conditioning (21%) than for the ATG regimen (48%). On the other hand, the 3-year moderate/severe chronic GvHD and overall survival rates were similar in both groups [24]. Reasons underlying these opposing results compared to our findings could be the small patient numbers and the inclusion of different hematological diseases as well as the unbalanced composition within the two groups (e.g. 6 ALL patients in the ATG group and 11 patients in the PTCy group).

A study by EBMT compared PTCy and ATG in a haploidentical setting in ALL patients and observed similar rates of acute and chronic GvHD, whereby the relapse incidence was higher in the ATG cohort. Moreover, in the PTCy cohort the authors observed improved LFS and OS [26]. Another study recently published by EBMT observed a reduced risk for extensive chronic GvHD and an inferior LFS as compared to the PTCy group in ALL patients receiving ATG and undergoing hematopoietic cell transplantation from a MUD [25]. For the whole cohort our results are in line with those of EBMT with no differences in overall survival or in the incidence of acute GvHD, but with a significantly higher incidence of chronic GvHD in the PTCy group as compared to ATG. Nevertheless, we observed after case matching (propensity score analysis) a trending higher incidence of aGvHD grades II-IV in the PTCy group compared to the ATLG group, with no significant differences observed in other transplant outcomes. To substantiate these findings a prospective study comparing PTCy and ATLG would be needed.

In contrast to the work by Giebel et al. with a significantly longer LFS at two years in the PTCy group, namely 71% vs 58% in the ATG group (p = 0.01), we observed no significant differences in LFS. The results concerning NRM in the work by Giebel et al. are in line with our results with no significant differences between the two study groups. Regarding cumulative incidence of relapse at two years, both Giebel et al. and we observed a higher rate in the ATG group than in the PTCy group. In contrast, on multivariate analysis in our calculation, the receipt of PTCy compared with ATLG was associated with a reduced relapse incidence [25]. With regard to toxicity profile, we observed in our study a higher rate of VOD, TMA, infections including CMV and cystitis for PTCy than for ATLG. Our results are in line with those of other groups, who also described a higher incidence of infections including cystitis when using PTCy as compared to ATG [25, 51, 52].

Currently, worldwide results for alloSCT high-risk ALL patients in CR1 are unsatisfying. An important factor should be improvement with long and deep suppression of disease-initiating clones until a graft-versus-leukemia effect can eradicate the underlying disease without adding toxicity. It is of tremendous importance to maintain a balance between immunosuppression and stimulation of the immune system in a way that allows an optimal GvL effect while avoiding excess organ damage.

This study is subject to several limitations, primarily stemming from its retrospective design. Notable among these is the missing data, which could impact the findings. In addition to variability in the conditioning regimens and the administered doses of ATLG, as well PTCy for GvHD prophylaxis starting in 2014, add to the complexity of our analysis. Additionally, the absence of the MRD status for numerous patients and the historical inconsistency in MRD assessment techniques further challenge the robustness of our results.

The intermittent use of TKIs, influenced by fluctuating availability, also poses a limitation. Evolutions in supportive care practices, assessment approaches for microangiopathy, the increasing preference for PBSC over the recent years, and the adoption of National Institutes of Health (NIH) criteria post-2005 are factors that may have bearing on our analysis.

Despite these challenges, we attempted to mitigate such limitations through rigorous multivariate analyses and propensity score matching. Nonetheless, gaps persist due to unavailable data on BCR-ABL status and MRD, and multicollinearity observed among variables such as ATLG versus PTCy, conditioning regimen, and year of transplant, which preclude a comprehensive resolution of all identified constraints.

In conclusion, our retrospective single-center analysis in a considerable number of patients shows that GvHD prophylaxis with PTCy in TBI based alloSCT for ALL lead to similar LFS and OS and may present adequate alternative immune suppression but probably lead to a higher incidence in acute and chronic GvHD and toxicity in comparison to ATLG.

Due to the heterogeneous cohorts, these data need to be validated in a prospective study.

### Supplementary information

Supplementary Figure 1

Supplementary Figure 2

Supplementary Figure 3

## Data Availability

The analyzed data are available from the corresponding author on reasonable request.
