# Supplementary figures and images for: Anti-T-lymphocyte globulin (ATLG) compared to post-transplant cyclophosphamide as GvHD prophylaxis in ALL patients undergoing allogeneic stem cell transplantation
Source: Bone Marrow Transplant. 2024 Jun 14;59(9):1265–74. doi: 10.1038/s41409-024-02328-w (PMC11368809; doi:10.1038/s41409-024-02328-w)

## Slide 1
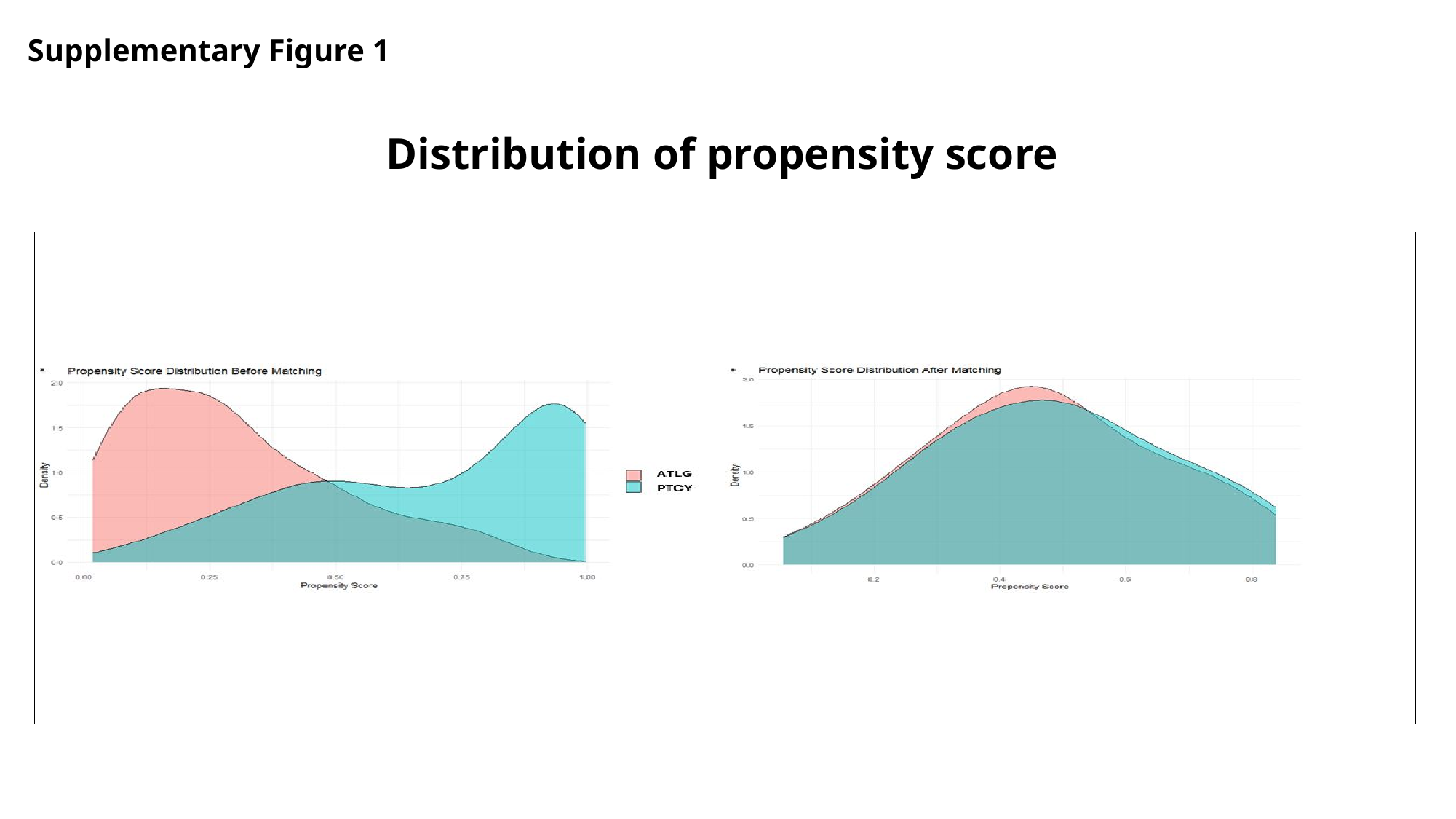

Supplementary Figure 1
Distribution of propensity score

Supplement: Supplementary file 1 — Supplementary Figure 1 [file 41409_2024_2328_MOESM1_ESM.pptx]

## Slide 1
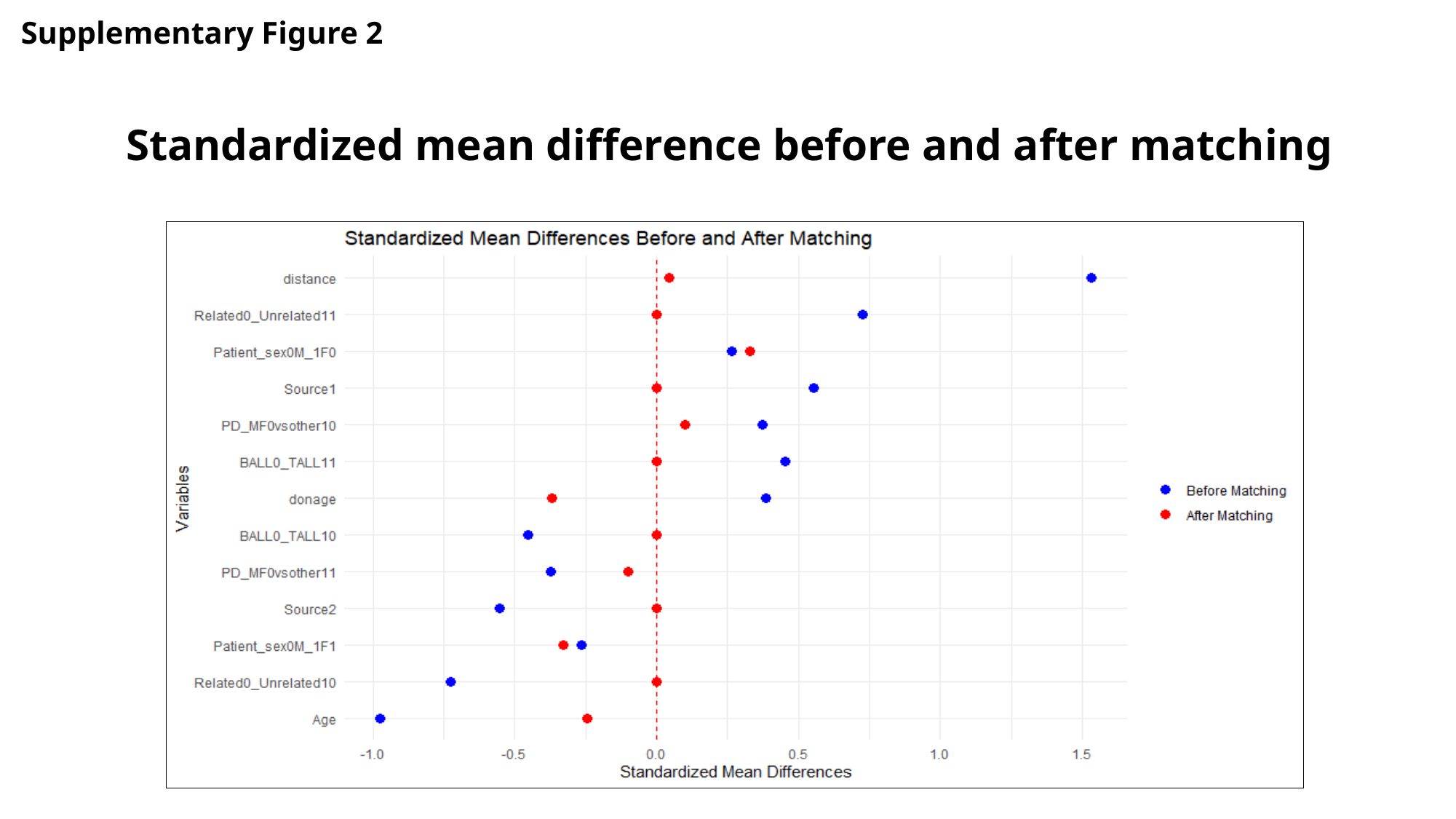

Supplementary Figure 2
Standardized mean difference before and after matching

Supplement: Supplementary file 2 — Supplementary Figure 2 [file 41409_2024_2328_MOESM2_ESM.pptx]
