# Supplementary material for: Anti-T-lymphocyte globulin (ATLG) compared to post-transplant cyclophosphamide as GvHD prophylaxis in ALL patients undergoing allogeneic stem cell transplantation
Source: Bone Marrow Transplant. 2024 Jun 14;59(9):1265–74. doi: 10.1038/s41409-024-02328-w (PMC11368809; doi:10.1038/s41409-024-02328-w)
Supplement: Supplementary file 3 — Supplementary Figure 3 [file 41409_2024_2328_MOESM3_ESM.pptx]

## Slide 1
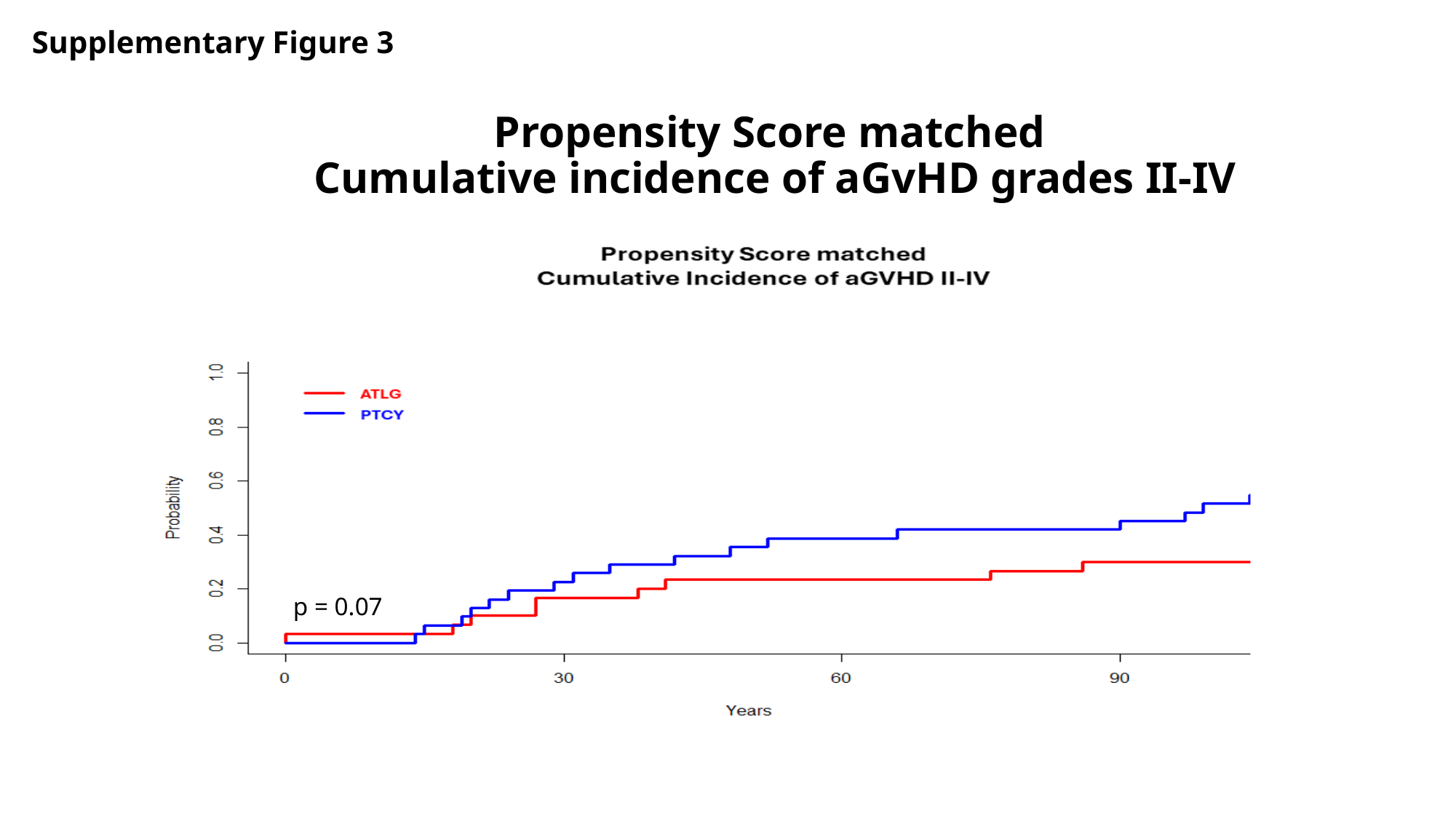

Supplementary Figure 3
Propensity Score matched
Cumulative incidence of aGvHD grades II-IV
p = 0.07
